# Neural correlates of implicit emotion regulation in mood and anxiety disorders: an fMRI meta-analytic review

**DOI:** 10.1038/s41598-025-03828-5

**Published:** 2025-06-04

**Authors:** Stefan Daniel Paul Dalton, Holly Cooper, Ben Jennings, Survjit Cheeta

**Affiliations:** 1 Centre for Cognitive and Clinical Neuroscience, Department of Life Sciences, College of Health, Medicine and Life Sciences, Brunel University London, Uxbridge, UB8 3PH UK; 2 Department of Psychology, Royal Holloway, University of London, Uxbridge, UK

**Keywords:** Implicit emotion regulation, Emotion regulation, Depression, Anxiety, Meta-analysis, fMRI, Emotion, Neuroscience, Diagnostic markers, Predictive markers, Prognostic markers, Psychiatric disorders

## Abstract

Maladaptive implicit emotion regulation has been highlighted as a transdiagnostic characteristic of mood and anxiety disorders. Whilst clinical diagnosis has relied on signs and symptoms, the integration of clinical neurosciences is becoming more important as a means of enhancing assessment, diagnosis, and treatment. Thus, activation likelihood estimation (ALE) meta-analysis was conducted for whole-brain foci comparing implicit emotion regulation in a large sample of patients with mood and anxiety disorders and healthy controls. Twenty-four clinical studies were identified based on established criteria (e.g., DSM-5). ALE meta-analysis reported convergence of hypoactivation in patients (*n* = 432) in the right medial frontal gyrus (BA9), spreading to the right anterior cingulate gyrus (BA32); and in the left middle temporal gyrus (BA21), spreading to the left superior temporal gyrus (BA22). Convergence of hyperactivation was reported in patients (*n* = 536) in the left medial frontal gyrus (BA9), spreading to the left superior frontal gyrus and the left middle frontal gyrus. Separate analysis of the mood disorders subgroup further highlighted convergence of hyperactivation in the insula and claustrum. The implications of the current findings are discussed within the context of the Research Domain Criteria (RDoC) framework of developing diagnostic systems that are more predictive of treatment outcomes.

**Supplementary Information:**

The online version contains supplementary material available at 10.1038/s41598-025-03828-5.

## Introduction

An inability to regulate emotions is a central characteristic of many psychiatric disorders. While previous research has largely focused on the role of maladaptive explicit (conscious) emotion regulation, where the goal is to alter the direction, intensity, or duration of an emotional experience, emotion regulation also occurs implicitly (unconsciously), running automatically without monitoring or effort, and can be activated without a conscious strategy, goal, or intent^1–10^. Importantly, our recent meta-analysis has highlighted the role of maladaptive implicit emotion regulation as a transdiagnostic characteristic of mood and anxiety disorders. It has also been suggested that maladaptive implicit emotion regulation may disrupt the effectiveness of conscious emotion regulation strategies through automatic bottom-up processes like the involuntary shifting of attention towards salient information i.e., rumination, worry, or negative automatic thoughts^11^.

In healthy individuals, the main neural regions recruited during explicit emotion regulation are the lateral regions of the prefrontal cortex (PFC), such as the ventrolateral prefrontal cortex (vlPFC) and dorsolateral prefrontal cortex (dlPFC)^12^. Studies investigating the neural underpinnings of maladaptive explicit emotional regulation in mood and anxiety disorders suggest the role of an extended network. In their meta-analysis, Gou et al.^13^ summarised findings from 28 fMRI studies (656 patients with depression and 680 healthy controls) on explicit emotion dysregulation and reported hyperactivity in the middle temporal gyrus (MTG), superior temporal gyrus (STG), parahippocampal gyrus, and cuneus in patients; regions which interact with the default mode network (DMN), which is known to play a role in self-referential processing, internally focused rumination, and negative cognitions^14,15^. In contrast, depressed patients also showed hypoactivity in the superior frontal gyrus (SFG), superior temporal gyrus, inferior parietal lobe, and insula; regions which interact with the salience network (SN), which is functional in the filtering of salient information and switching from the DMN to the executive control network, known for its role in higher cognitive processes^16^. The involvement of a broad spectrum of neural regions was also the main finding of a systematic review of 17 neuroimaging studies (528 patients and 403 healthy controls) on functional connectivity in depressed patients while undertaking an emotional face processing task. Compared to the healthy controls, the processing of negatively valanced faces was associated with reduced effective connectivity from the dlPFC to the amygdala, whereas the processing of happy facial expressions was associated with greater inhibitory connectivity from the ventromedial prefrontal cortex (vmPFC) to the amygdala^17^. Finally, a meta-analysis of 23 fMRI studies (449 patients and 424 healthy controls) examining disrupted neural facial processing in social anxiety disorder (SAD) reported several altered activations^18^. Whilst supporting the large body of existing evidence on the role of bilateral amygdala hyperactivity in SAD, increased activation was also seen in areas related to attention (SFG), emotional recognition (superior temporal sulcus), emotion regulation (medial frontal gyrus and subgenual anterior cingulate) and the visual cortex. In contrast, areas that were more active in healthy controls were clusters in the occipital visual cortex (lingual gyrus) and the posterior cingulate.

In healthy individuals, neural regions known to be recruited during implicit emotion regulation are the medial regions of the PFC, including the vmPFC and the anterior cingulate cortex (ACC)^12^. In patients with mood and anxiety disorders, several neural regions have been identified within the literature, although there are discrepancies regarding the precise regions implicated in implicit emotion regulation. For instance, the vmPFC^19,20^, dorsomedial prefrontal cortex (dmPFC)^21,22^, vlPFC, dlPFC^19,22–25^, the orbitofrontal cortex (OFC)^25,26^, the ACC^23,27^, the insula^19,23,28^, and the amygdala^19,20,24,25,29–34^ have been identified during implicit emotion regulation. Furthermore, the neural activation in patients during implicit emotion regulation have reported both increased and decreased activation within those regions^19,24,29,31,33,35^, with many studies being limited by investigation of an isolated region of interest (ROI) determined by a priori hypotheses, which may introduce bias into the literature.

Therefore, the aim of the present activation likelihood estimation (ALE) meta-analysis was to examine neural regions activated during implicit emotion regulation by directly comparing mood and anxiety disorder patients to healthy controls. The findings will offer support to the National Institute of Mental Health (NIHM) Research Domain Criteria (RDoC) framework, where the aim is to develop diagnostic systems that rely on both clinical observations and recent research developments in the clinical neurosciences^36^. Understanding of the neural underpinnings of implicit emotion regulation in mood and anxiety disorders may not only reveal new insights into their pathophysiology and diagnosis, but also predict better treatment outcomes^12,36–39^.

## Results

### Study selection and data extraction

**Fig. 1:**
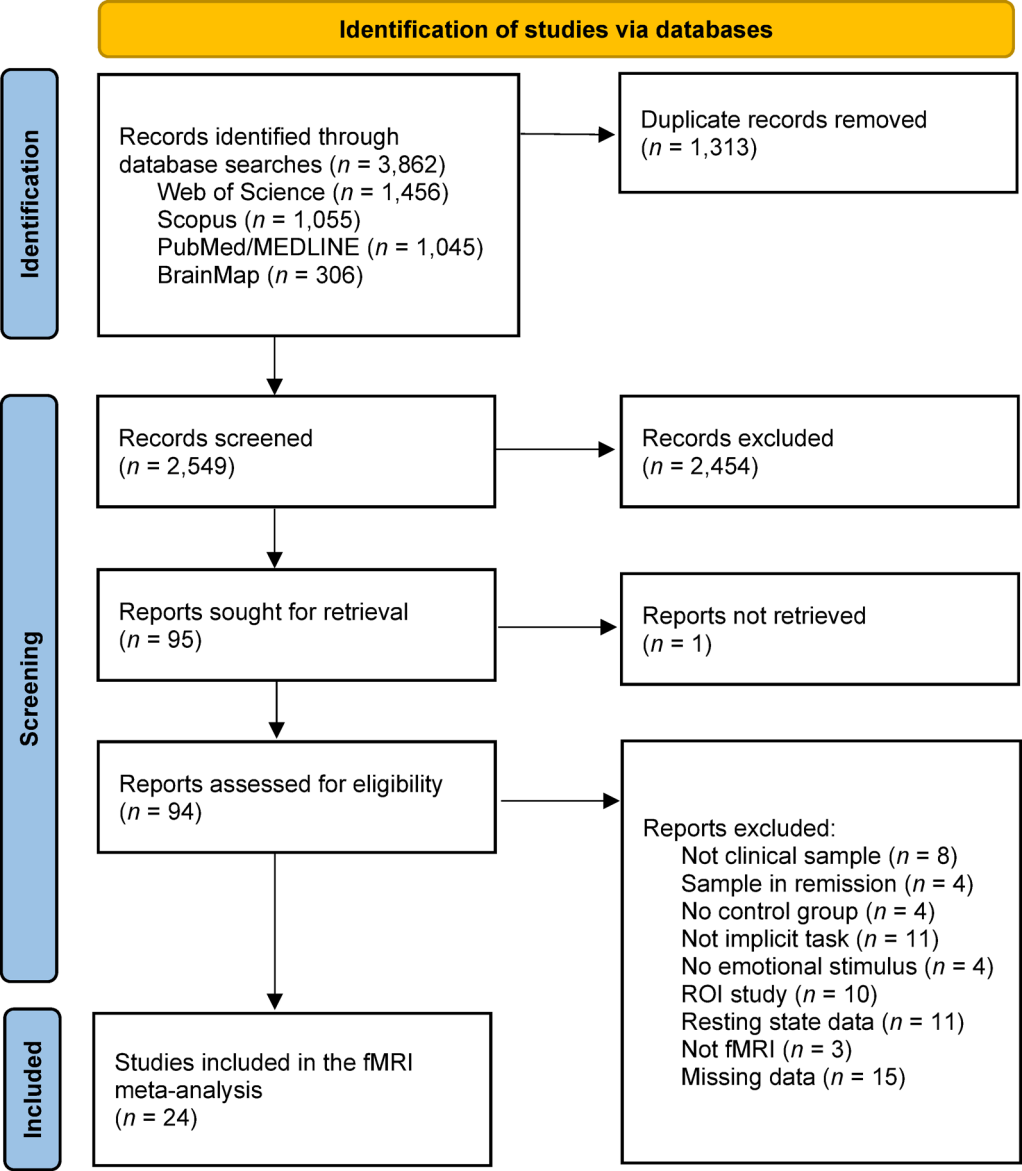
PRISMA flowchart of study selection process. *Note.* Preferred Reporting Items for Systematic Reviews and Meta-Analyses (PRISMA) flowchart of the study selection process^40^*.*

Articles were sought after in the Web of Science (*n* = 1,456), Scopus (*n* = 1,055), PubMed/MEDLINE (*n* = 1,045), and BrainMap (*n* = 306) databases. The OpenGrey database produced no records. Duplicate records were removed, and the titles and abstracts of the remaining articles were assessed against eligibility criteria, with a total 2,454 records failing to meet inclusion. The final articles (*n* = 95) were retrieved for full-text screening. Full-text screening resulted in the exclusion of further studies due to several reasons including the sample not meeting a clinical diagnosis or being in remission, no healthy control comparison sample, not investigating implicit emotion regulation, not evaluating responses to emotional stimuli, a ROI study, resting state data, not an fMRI design, and missing data/no access. Authors were contacted to manage missing data, with one response. A final list of neuroimaging studies (*n* = 24) were eligible for a coordinate-based meta-analysis. The full study selection details are presented in a PRISMA flowchart^40^ in Fig. 1. A full PRISMA checklist is available in Appendix A of *Supplementary Materials.*

Of the 24 studies (patients: *n* = 684; healthy controls: *n* = 579) included in the ALE meta-analysis, eight were major depressive disorder/unipolar depression^29,31,37,41–45^, two were bipolar disorder^37,41^, five were generalised anxiety disorder^33,42,46–48^, six were generalised social phobia/social anxiety disorder^27,35,46,49–51^, six were panic disorder with/without agoraphobia^21,22,32,38,47,52^, and three were post-traumatic stress disorder^47,53,54^. Note that some studies examined more than one psychiatric disorder. Implicit emotion regulation paradigms extracted from the literature searches included implicit emotional response inhibition, implicit attentional cognitive control, implicit cognitive reappraisal, implicit emotional conflict adaptation, implicit emotional regulation, attentional performance with emotional distractors, indirect emotional facial processing, directed attention with disorder specific stimuli, and emotional-associative learning. Characteristics of the studies included in the ALE meta-analysis can be viewed in Table 1.

**Table 1:** Characteristics of mood and anxiety disorder studies included in the activation likelihood estimation meta-analysis.

Author(s)	Sample	Psychiatric disorder(s)	Diagnostic measurement	Comorbidities	Implicit emotion regulation study characteristics
Wang et al., 2021	26 patients, 25 controls	Panic disorder	DSM-5	Generalised anxiety disorder, social anxiety disorder, depressive disorder	PD and healthy control participants view negative images preceded by either a negative or non-negative description. Neural activation is observed during the presentation of negative images comparatively between the two (preceding negative and non-negative description) conditions.
Thomaes et al., 2012	29 patients, 22 controls	Post-traumatic stress disorder	The Structured Clinical Interview for DSM-IV-TR Axis I disorders, the Structured Clinical Interview for Disorders of Extreme Stress Not Otherwise Specified & the Clinician Administered PTSD Scale	Anxiety-disorders, depressive disorders, personality disorders	Implicit emotional conflict regulation in PTSD and healthy control participants. On high emotional conflict *(i.e.*,* incongruent [I])* trials, participants must respond to the colour of negative and trauma-related affect labels, irrespective of the affect label which are ‘task-irrelevant’.
Yu et al., 2015	19 patients, 19 controls	Generalised anxiety disorder	DSM-IV	*n/a*	Response inhibition in GAD and healthy controls towards negative stimuli. Participants respond to the biological sex of faces (Go/No-Go) irrespective of sad emotional facial expressions, which are ‘task-irrelevant’.
Etkin & Schatzberg, 2011	57 patients, 32 controls	Generalised anxiety disorder & major depressive disorder	Mini International Neuropsychiatric Interview	Comorbid generalised anxiety disorder & major depressive disorder	Implicit emotional conflict regulation in MDD, GAD, and healthy control participants. Participants must categorise emotional facial expressions, irrespective of overlaid (‘fear’ and ‘happy’) affect labels which are ‘task-irrelevant’.
Heitmann et al., 2017	24 patients, 24 controls	Generalised social anxiety disorder	Structured Clinical interview for DSM-IV	Major depressive disorder, specific phobia, obsessive-compulsive disorder, general anxiety disorder	SAD and healthy control participants respond to a bar orientation task, irrespective of disorder-related (e.g., giving speech, discussion scene, job interview) scenes.
Palm et al., 2011	15 patients, 16 controls	Generalised anxiety disorder	Structured Clinical Interview for DSM-IV Disorders	Social phobia, specific phobia, panic attacks	GAD and healthy control participants are presented with anger, disgust, fear, happiness, and sadness emotional facial expressions and must identify the biological sex of the face.
Bürger et al., 2017	72 patients, 36 controls	Unipolar depression & bipolar disorder	The Structured Clinical Interview for DSM-IV Axis I Disorders	Panic disorder, agoraphobia, generalised anxiety disorder, social phobia, specific phobia, obsessive-compulsive disorder, post-traumatic stress disorder, somatoform disorder, eating disorder, dysthymia, alcohol abuse, substance abuse	MDD, BD and healthy control participants must recognise and match facial stimuli, irrespective of angry, fearful, and happy facial expressions, that are ‘task-irrelevant’.
Arnone et al., 2012	38 patients, 54 controls	Major depressive disorder	Structured Clinical Interview for DSM-IV Axis I Disorders	*n/a*	MDD and healthy control participants identify the biological sex of sad, fearful, and happy emotional facial expressions, irrespective of the emotional content which is ‘task-irrelevant’.
Klumpp et al., 2013	29 patients, 27 controls	Generalised social anxiety disorder	Structured Clinical Interview for DSM-IV	*n/a*	gSAD and healthy control participants match geometric shapes and angry, fearful and happy emotional faces.
Gaebler et al., 2013	21 patients, 21 controls	Social anxiety disorder	Structured Clinical Interview for DSM-IV Axis I Disorders	Major depression, panic disorder, obsessive-compulsive disorder, dysthymia	SAD and healthy control participants match geometric shapes and faces with the presentation of angry and fearful emotional faces, which are ‘task irrelevant’.
Cerullo et al., 2014	50 patients, 25 controls	Major depressive disorder, bipolar disorder	Structured Clinical Interview for DSM-IV Axis I Disorders	Panic disorder, post-traumatic stress disorder, generalised anxiety disorder, obsessive-compulsive disorder, social anxiety disorder, substance use	MDD, BP-I, and healthy control participants complete an attentional performance task by responding to circles with the inclusion of distractor unpleasant emotional scenes.
Kraus et al., 2018	14 patients, 12 controls	Social anxiety disorder	Structured Clinical Interview for DSM-IV Disorders	Obsessive-compulsive disorder, specific phobia	SAD and healthy control participants view fearful emotional facial expressions and respond to the biological sex.
Blair et al., 2011	25 patients, 23 controls	Social anxiety disorder	Structured Clinical interview for DSM-IV Axis I disorders	*n/a*	SAD and healthy control participants identify the biological sex of morphing fearful, angry, and happy emotional facial expressions across various intensities.
Schwarzmeier et al., 2019	10 patients, 10 controls	Panic disorder	DSM-IV-TR	Unipolar depression, anxiety disorders	PD and healthy control participants complete an agoraphobia symptom provocation task in which biological male facial stimuli are presented with an aversive panic scream.
Mazza et al., 2012	10 patients, 10 controls	Post-traumatic stress disorder	Clinician-Administered PTSD Scale for DSM-IV criteria	*n/a*	PTSD and healthy control participants are presented with happy and sad emotional facial expressions that are followed by ideographs, which must be judged on pleasantness.
Korgaonkar et al., 2021	22 patients, 33 controls	Panic disorder	Mini International Neuropsychiatric Interview using DSM-IV criteria	Generalised anxiety disorder, major depressive disorder, obsessive-compulsive disorder, social phobia, agoraphobia	Implicit processing of sad, fear, anger, disgust, and happy emotional facial expressions in PD and healthy control participants.
Blair et al., 2012	50 patients, 18 controls	Generalized anxiety disorder & generalised social phobia	Structural Clinical interview for DSM-IV Axis I disorders	Comorbid generalised anxiety disorder & generalised social phobia	Emotional attention regulation in GAD, SAD, and healthy control participants. Participants are required to complete a number matching task irrespective of the presentation of positive and negative images that are ‘task-irrelevant’.
Neumeister et al., 2018	60 patients, 60 controls	Panic disorder, generalised anxiety disorder, post-traumatic stress disorder	Structured Clinical Interview for DSM-IV	Depressive disorder, specific phobia, social anxiety disorder, agoraphobia, eating disorder, somatoform disorder	Comparing the implicit processing of fearful facial stimuli between PD, GAD, PTSD, and healthy control participants.
Mitterschiffthaler et al., 2008	17 patients, 17 controls	Major depressive disorder	Structured Clinical interview for DSM-IV Axis I disorders	*n/a*	MDD and healthy control participants are presented with negative words in red, blue, green, or yellow and must respond to the colour of the negative words.
Ruhé et al., 2011	22 patients, 22 controls	Major depressive disorder	Structured Clinical interview for DSM-IV Axis I disorders	Anxiety disorders, substance use	MDD and healthy control participants are presented with fearful, angry, and happy emotional facial stimuli. Participants must make judgements based on the biological sex of the faces.
Frodl et al., 2009	12 patients, 12 controls	Major depressive disorder	DSM-IV	*n/a*	MDD and healthy control participants are presented with a trio of sad and angry faces. Participants must match the biological sex of faces with a target face.
Kaldewaij et al., 2019	18 patients, 17 controls	Panic disorder with/without agoraphobia	Structured Clinical Interview for DSM-IV Axis I Disorders	Agoraphobia, specific phobia	PD and healthy control participants identify the biological sex of morphing fearful and happy emotional facial expressions across various intensities irrespective of the emotional content.
Feldker et al., 2018	26 patients, 26 controls	Panic disorder with/without agoraphobia	Structured Clinical Interview for DSM-IV Axis I Disorders	Agoraphobia, depression, comorbid generalised anxiety disorder, somatic symptom disorder, social phobia, obsessive-compulsive disorder, bulimia nervosa	PD and healthy control participants respond to a bar orientation task, irrespective of panic-related (e.g., chest pain, hyperventilation, crowded areas) scenes.
Chechko et al., 2013	18 patients, 18 controls	Major depressive disorder	DSM-IV Structured Clinical Interview	*n/a*	Implicit emotional conflict regulation in MDD and healthy control participants. On high emotional conflict *(incongruent [I])* trials, participants must respond to the emotional facial expression irrespective of the overlaid affect labels which are ‘task-irrelevant’.

Note. Characteristics of mood and anxiety disorder studies (*n* = 24) investigating implicit emotion regulation included in the meta-analysis. Table reports author(s) and year of publication, sample size, psychiatric disorder(s) [major depressive disorder/unipolar depression, bipolar disorder, generalised anxiety disorder, generalised social phobia/social anxiety disorder, panic disorder with/without agoraphobia, and post-traumatic stress disorder], diagnostic criteria [the Structured Clinical Interview for the Diagnostic and Statistical Manual Of Mental Disorders (DSM), the Structured Clinical Interview for Disorders of Extreme Stress Not Otherwise Specified, the International Classification of Diseases (ICD), and the Mini International Neuropsychiatric Interview], comorbidities, and study characteristics. *GAD* = generalised anxiety disorder; *MDD* = major depressive disorder; *BP-I* = bipolar disorder; *SAD* = social anxiety disorder; *gSAD* = generalised social anxiety disorder; *PD* = panic disorder; *PTSD* = post-traumatic stress disorder. Under comorbidities *n/a* represents either no comorbidities or unreported comorbidities.

## Quality assessment

To assess the quality of studies, the Newcastle-Ottawa Scale adapted for cross-sectional studies^55^ was modified for the purpose of this review. The outcome reported that 22 studies were of excellent quality and two were of good quality (see Table 2). The full list of assessment criteria (Appendix B) along with the list of corresponding authors of Table 2 (Appendix C) can be found in the *Supplementary Materials.*

**Table 2:** Quality of mood and anxiety disorder studies included in the activation likelihood estimation meta-analysis.

Author			[1]	[2]	[3]	[4]	[5]	[6]	[7]	[8]	[9]	[10]	[11]	[12]	[13]	[14]	[15]	[16]	[17]	[18]	[19]	[20]	[21]	[22]	[23]	[24]
Selection: (Maximum 4 stars)	(1)	Representativeness of the clinical sample	*	*	*	*	*	*	*	*	*	*	*	*	*	*	*	*	*	*	*	*	*	*	*	*
	(2)	Selection of the non-clinical sample	*	*	*	*	*	*	*	*	*	*	*	*	*	*	*	*	*	*	*	*	*	*	*	*
	(3)	Ascertainment of exposure	*	**	**	**	**	*	**	**	**	**	**	**	**	*	**	**	**	*	*	**	**	**	**	**
	(4)	Demonstration that outcome of interest was not present in non-clinical sample	*	*	*	*	*	*	*	*	*	*	*	*	*	*	*	*	*		*	*	*	*	*	*
Comparability (Maximum 2 stars)	(5)	Comparability of samples in the different outcome groups based on design or analysis.	**	*	**	**	**	**	**	**	**	**	**	**	**	**	**	**	**	**	**	**	**	**	**	**
Outcome: (Maximum 3 stars)	(6)	Assessment of outcome	**	**	*	*	*	**	*	*	**	**	*	*	*	**	*	*	**	*	*	*	**	**	**	*
	(7)	Statistical test	*	*	*	*	*	*	*	*	*	*	*	*	*	*	*	*	*	*	*	*	*	*	*	*
		Total score	9	9	9	9	9	9	9	9	10	10	9	9	9	9	9	9	10	7	8	9	10	10	10	9

Notes. Results of the Newcastle-Ottawa Scale adapted for cross-sectional studies^55^ reported that 22 studies were of excellent quality and two were of good quality. Full quality assessment criteria (Appendix B) and the list of corresponding authors (Appendix C) can be viewed in the *Supplementary Materials.*

### Activation likelihood estimation meta-analysis

Activation likelihood estimation meta-analyses were conducted for mood and anxiety disorders combined. For exploratory purposes, subgroup analyses were performed for mood and anxiety disorders separately. All significant results for whole-brain foci during implicit emotion regulation are reported in Table 3.

**Table 3:** Significant clusters of activation likelihood estimation meta-analysis for whole-brain foci in mood and anxiety disorder patients and healthy controls during implicit emotion regulation at FWE *p* <.05.

Anatomical region	Brodmann area	Side		Cluster size	Peak coordinates MNI			ALE score
					***x***	***y***	***z***	
Mood and anxiety disorders								
*Hypoactivation in patients* (controls > patients)								
Medial frontal gyrus, anterior cingulate gyrus	9, 32	R		928 mm^3^	4	38	26	0.023
Middle temporal gyrus, superior temporal gyrus	21, 22	L		1,216 mm^3^	−64	−2	−14	0.020
					−56	−6	−20	0.017
*Hyperactivation in patients* (patients > controls)								
Medial frontal gyrus, superior frontal gyrus, middle frontal gyrus	9, 10	L	736 mm^3^		−22	50	12	0.023
Mood disorders								
*Hypoactivation in patients* (controls > patients)								
Medial frontal gyrus, anterior cingulate gyrus	9, 32	R, L		1,128 mm^3^	4	38	26	0.023
Middle temporal gyrus, superior temporal gyrus	21, 22	L		1440 mm^3^	−64	−2	−14	0.020
Parahippocampal gyrus, culmen	36, 35	L		648 mm^3^	−26	−34	−18	0.018
*Hyperactivation in patients* (patients > controls)								
Medial frontal gyrus, superior frontal gyrus, middle frontal gyrus	9, 10	L		1008 mm^3^	−22	50	12	0.023
Orbital gyrus	47, 11	R		872 mm^3^	20	20	−32	0.020
Inferior frontal gyrus	44, 45	L		512 mm^3^	−54	22	10	0.016
Middle frontal gyrus	47	L		552 mm^3^	−46	40	−8	0.017
Medial frontal gyrus	6	R, L		520 mm^3^	4	−2	52	0.017
Anterior cingulate gyrus, cingulate gyrus	32, 24, 33	L		752 mm^3^	0	26	16	0.017
Insula	13	R		536 mm^3^	42	2	14	0.017
Lentiform nucleus (putamen)		L		616 mm^3^	−26	−6	6	0.017
Claustrum		L		504 mm^3^	−38	−14	−4	0.016
Claustrum, insula		R		536 mm^3^	42	−14	−8	0.017
Anxiety disorders								

No significant clusters reported.

Table 3. Significant areas of convergence of hypoactivation and hyperactivation for whole-brain foci in mood and anxiety disorder patients during implicit emotion regulation. A cluster-level family-wise error correction threshold of *p* <.05 was used, with an uncorrected value of *p* <.001. A rigorous threshold of 5,000 permutations was applied. Peak coordinates are reported. Locations are reported to be 100% grey matter.

### Convergence of hypoactivation in mood and anxiety disorders during implicit emotion regulation

**Fig. 2:**
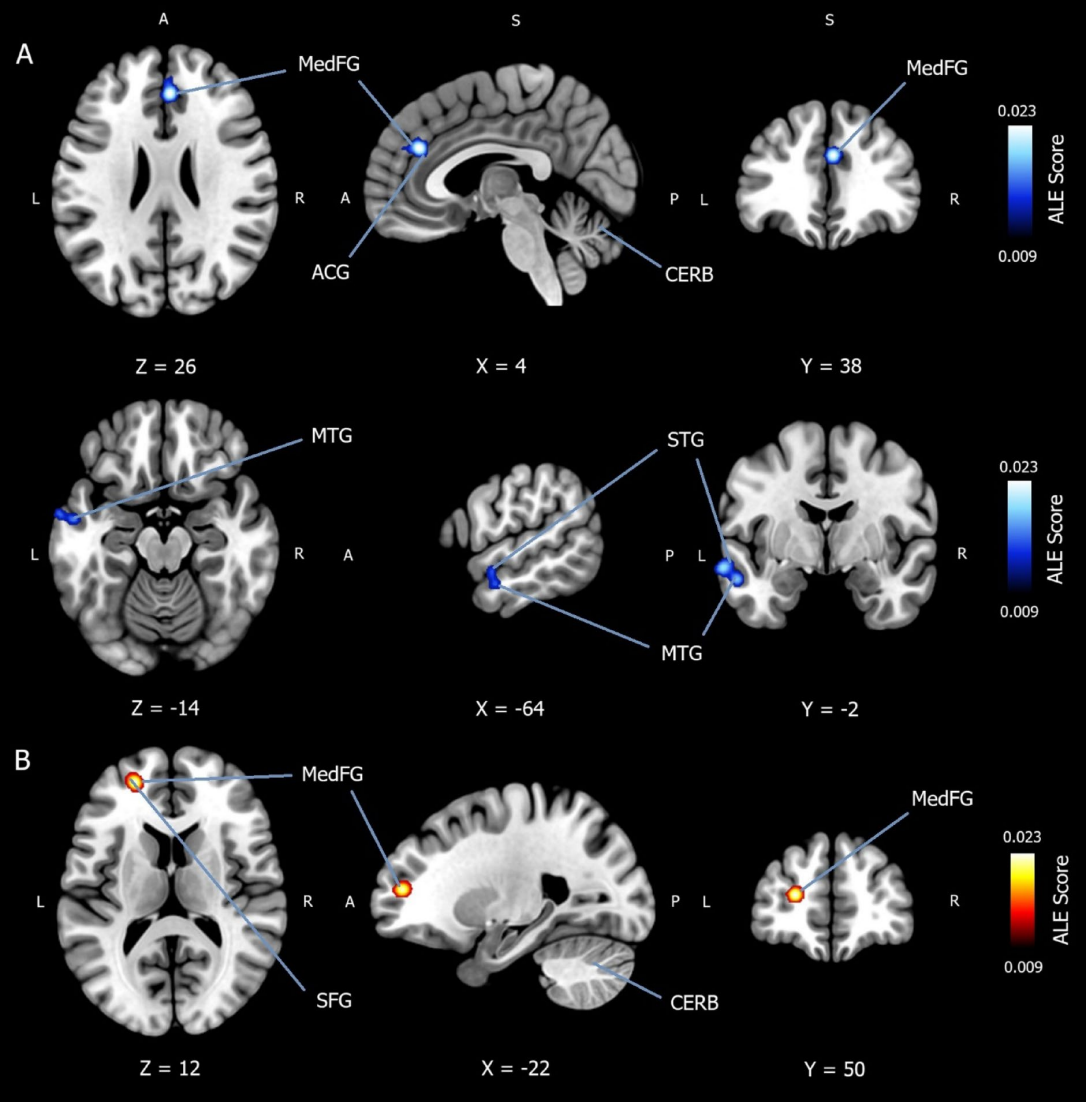
Activation likelihood estimation meta-analysis for convergence of hypoactivation and hyperactivation in mood and anxiety disorder patients during implicit emotion regulation. (**A**) Activation likelihood estimation (ALE) meta-analysis (*n* = 432) showing convergence of hypoactivation in patients compared to healthy controls (controls > patients) during implicit emotion regulation. Two cluster regions with three peak coordinates were identified using a cluster-level family-wise error (FWE) correction threshold of *p* < .05, with an uncorrected value of *p* <.001. A rigorous threshold of 5,000 permutations was applied. The first cluster reported a peak coordinate (x = 4, y = 38, z = 26) in the right medial frontal gyrus (BA9), extending over the right anterior cingulate gyrus (BA32). The second cluster yielded two peak coordinates ([x = −64, y = −2, z = −14] and [x = −56, y = −6, z = −20]) in the left middle temporal gyrus (BA21), extending over the left superior temporal gyrus (BA22). All locations were reported to be 100% grey matter. *MedFG* = medial frontal gyrus; *SFG* = superior frontal gyrus; *ACG* = anterior cingulate gyrus; *MTG* = middle temporal gyrus; *STG* = superior temporal gyrus; *CERB* = cerebellum; *ALE Score* = ALE value. (**B**) ALE meta-analysis (*n* = 536) showing convergence of hyperactivation in mood and anxiety disorder patients compared to healthy controls (patients > controls) during implicit emotion regulation. One cluster region with one peak coordinate was identified using a cluster-level FWE correction threshold of *p* < .05, with an uncorrected value of *p* < .001. A rigorous threshold of 5,000 permutations was applied. The peak coordinate (x = −22, y = 50, z = 12) was reported in the left medial frontal gyrus (BA9), spreading to both the left superior frontal gyrus (BA10) and the left middle frontal gyrus (BA10). All locations were reported to be 100% grey matter.

In total, 432 patients were included in the ALE meta-analysis, which converged whole-brain foci for decreased activation in mood and anxiety disorders (controls > patients) during implicit emotion regulation. A total of *k* = 19 experiments were extracted from the included studies and can be viewed in Appendix E of the *Supplementary Materials*. Six studies were MDD^29,31,37,41,42,45^, one was BP^41^, four were GAD^33,42,46,48^, three were SAD^46,49,51^, three were PD^22,38,52^, and one was PTSD^53^. Note that some studies examined more than one psychiatric disorder. A cluster-level family-wise error (FWE) correction threshold of *p* <.05 was used, with an uncorrected value of *p* <.001. A rigorous threshold of 5,000 permutations was applied. The ALE meta-analysis reported significantly more convergence of hypoactivation (controls > patients) in two cluster regions with three peak coordinates. The first cluster reported a peak coordinate (*x* = 4, *y* = 38, *z* = 26) in the right medial frontal gyrus (BA9), extending over the right anterior cingulate gyrus (BA32). The second cluster yielded two peak coordinates ([*x* = −64, *y* = −2, *z* = −14] and [*x* = −56, *y* = −6, *z* = −20]) in the left MTG (BA21), extending over the left STG (BA22). All locations were reported to be 100% grey matter. The result is available in Table 1. And can be viewed in Fig. 2A.

### Convergence of hyperactivation in mood and anxiety disorders during implicit emotion regulation

In total, 536 patients were included in the ALE meta-analysis, which converged whole-brain foci for increased activation in mood and anxiety disorders (patients > controls) during implicit emotion regulation. A total of *k* = 24 experiments were extracted from the included studies and can be viewed in Appendix F of the *Supplementary Materials*. Seven studies were MDD^29,31,41–45^, one was BP^41^, four were GAD^42,46–48^, six were SAD^27,35,46,49–51^, five were PD^21,32,38,47,52^, and three were PTSD^47,53,54^. Note that some studies investigated multiple psychiatric disorders. A cluster-level FWE correction threshold of *p* <.05 was used, with an uncorrected value of *p* <.001. A rigorous threshold of 5,000 permutations was applied. The ALE meta-analysis reported significantly more convergence of hyperactivation (patients > controls) in one cluster region with one peak coordinate. The peak coordinate (*x* = −22, *y* = 50, *z* = 12) was reported in the left medial frontal gyrus (BA9), spreading to both the left SFG (BA10) and left middle frontal gyrus (MFG) (BA10). All locations were reported to be 100% grey matter. The result is available in Table 1. And can be viewed in Fig. 2B.

### Subgroup analysis for convergence of hypoactivation in mood disorders during implicit emotion regulation

**Fig. 3:**
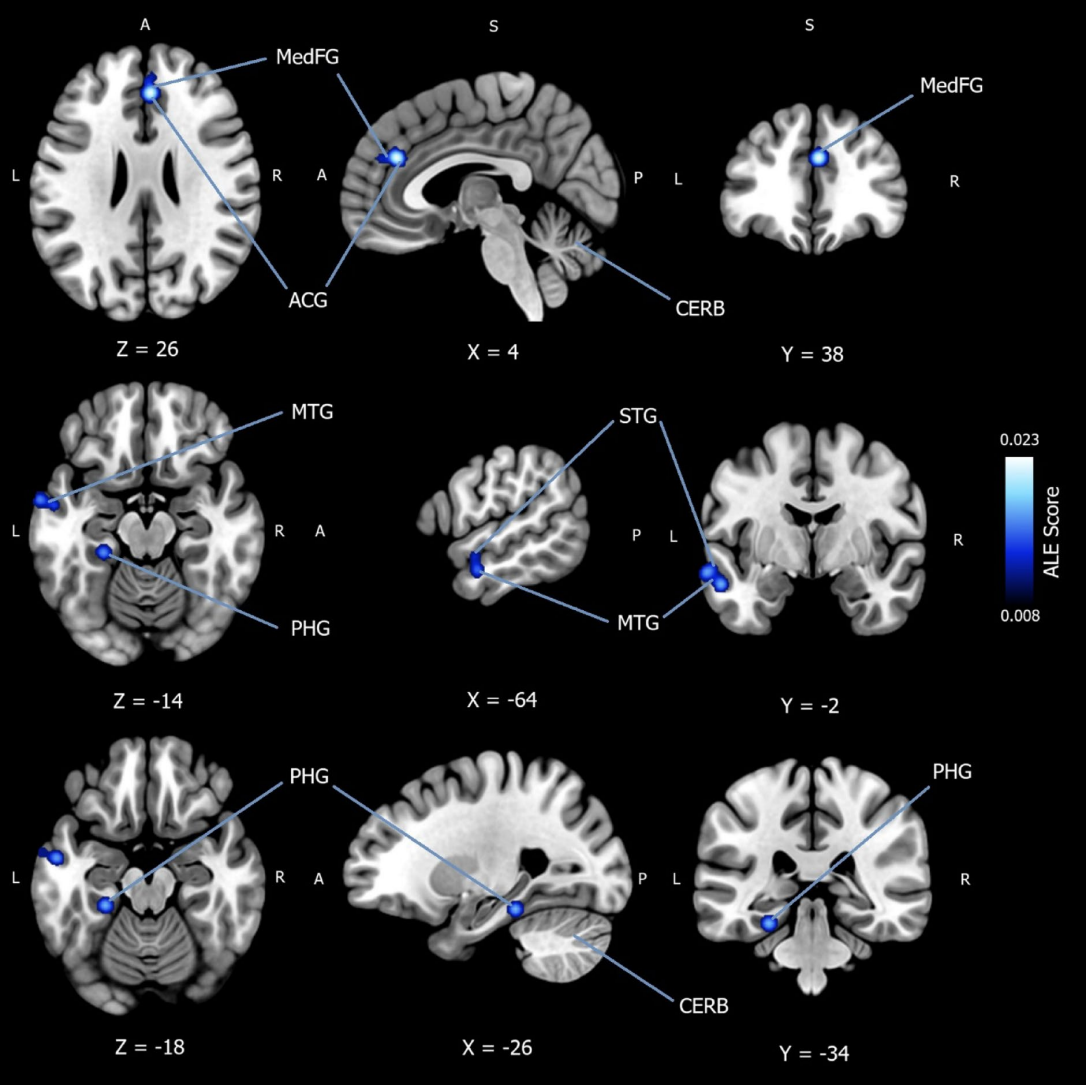
Subgroup analysis for convergence of hypoactivation in mood disorder patients during implicit emotion regulation. Activation likelihood estimation meta-analysis (*n* = 225) showing convergence of hypoactivation in mood disorder patients compared to healthy controls (patients > controls) during implicit emotion regulation. Three cluster regions with five peak coordinates were identified using a cluster-level family-wise error correction threshold of *p* < .05, with an uncorrected value of *p* < .001. A rigorous threshold of 5,000 permutations was applied. The first cluster reported a peak coordinate (x = 4, y = 38, z = 26) in the right and left medial frontal gyrus (BA9), extending over the anterior cingulate gyrus (BA32). The second cluster yielded two peak coordinates ([x = −64, y = −2, z = −14] and [x = −56, y = −6, z = −20]) in the left middle temporal gyrus (BA21), extending over the left superior temporal gyrus (BA22). Finally, two peak coordinates ([x = −26, y = −34, z = −18] and [x = −16, y = −38, z = −20]) were reported in the parahippocampal gyrus (BA36, BA35), spreading over the culmen. All locations were reported to be 100% grey matter. *MedFG* = medial frontal gyrus; *ACG* = anterior cingulate gyrus; *MTG* = middle temporal gyrus; *STG* = superior temporal gyrus; *PHG* = parahippocampal gyrus; *CERB* = cerebellum; *ALE Score* = ALE value.

In total, 207 patients were included in the ALE meta-analysis, which converged whole-brain foci for decreased activation in mood disorders (controls > patients) during implicit emotion regulation. A total of *k* = 8 experiments were extracted from the included studies and can be viewed in Appendix G of the *Supplementary Materials*. Six studies were MDD^29,31,37,41,42,45^, and one was BP^41^. A cluster-level FWE correction threshold of *p* <.05 was used, with an uncorrected value of *p* < .001. A rigorous threshold of 5,000 permutations was applied. The ALE meta-analysis reported significantly more convergence of activation (controls > patients) in three cluster regions with five peak coordinates. The first cluster reported a peak coordinate (*x* = 4, *y* = 38, *z* = 26) in the right and left medial frontal gyrus (BA9), extending over the anterior cingulate gyrus (BA32). The second cluster yielded two peak coordinates ([*x* = −64, *y* = −2, *z* = −14] and [*x* = −56, *y* = −6, *z* = −20]) in the left MTG (BA21), extending over the left STG (BA22). Finally, two peak coordinates ([*x* = −26, *y* = −34, *z* = −18] and [*x* = −16, *y* = −38, *z* = −20]) were reported in the parahippocampal gyrus (BA36, BA35), spreading over the culmen. All locations were reported to be 100% grey matter. The result is available in Table 1. And can be viewed in Fig. 3.

## Subgroup analysis for convergence of hyperactivation in mood disorders during implicit emotion regulation

**Fig. 4:**
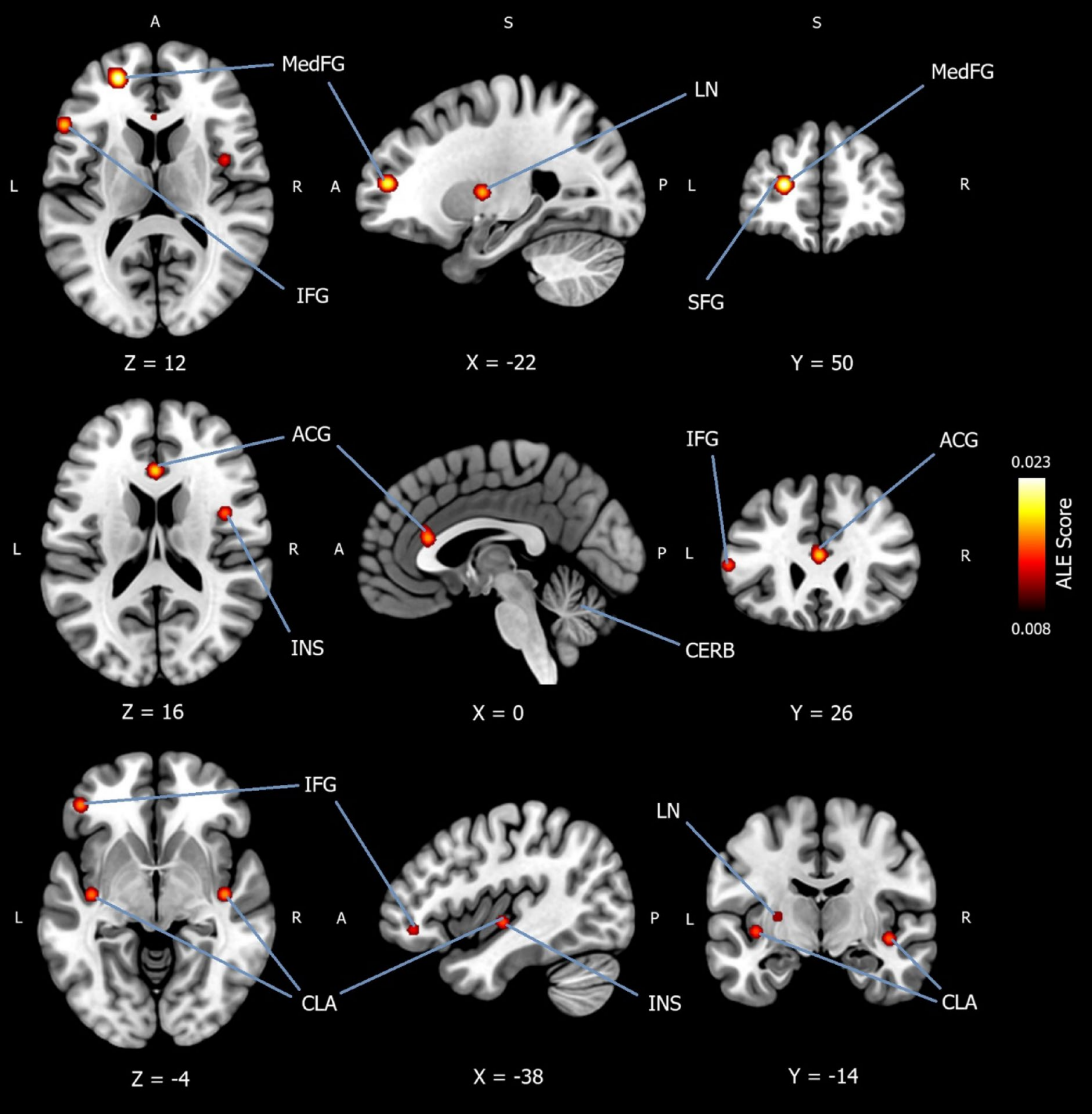
Subgroup analysis for convergence of hyperactivation in mood disorder patients during implicit emotion regulation. Activation likelihood estimation meta-analysis (*n* = 225) showing convergence of hyperactivation in mood disorder patients compared to healthy controls (patients > controls) during implicit emotion regulation. Ten cluster regions with ten peak coordinates were identified using a cluster-level family-wise error correction threshold of *p* < .05, with an uncorrected value of *p* < .001. A rigorous threshold of 5,000 permutations was applied. The first peak coordinate (x = −22, y = 50, z = 12) was reported in the left medial frontal gyrus (BA9), spreading to the left superior frontal gyrus (BA10), and left middle frontal gyrus (BA10). Peak coordinates were reported in the right orbital gyrus (x = 20, y = 20, z = −32) (BA47, BA11) and in the left (x = −54, y = 22, z = 10) inferior frontal gyrus (BA44, BA45). Peak coordinates (x = −46, y = 40, z = −8) were reported in the left middle frontal gyrus (BA47) and in the right and left (x = 4, y = −2, z = 52) medial frontal gyrus (BA6). Peak coordinates (x = 0, y = 26, z = 16) were reported in the left anterior cingulate gyrus (BA32), spreading to the left cingulate gyrus (BA24, BA33). A peak coordinate (x = 42, y = 2, z = 14) was reported in the right insula (BA13) and in the left lentiform nucleus (putamen) (x = −26, y = −6, z = 6). Finally, peak coordinates were reported in left claustrum (x = −38, y = −14, z = −4) and the right claustrum (x = 42, y = −14, z = −8), spreading to the insula (BA13). All locations were reported to be 100% grey matter. *MedFG* = medial frontal gyrus; *SFG* = superior frontal gyrus; *IFG* = inferior frontal gyrus; *ACG* = anterior cingulate gyrus; *INS* = insula; *LN* = lentiform nucleus; *CLA* = claustrum; *CERB* = cerebellum; *ALE Score* = ALE value.

In total, 225 patients were included in the ALE meta-analysis, which converged whole-brain foci for increased activation in mood disorders (patients > controls) during implicit emotion regulation. A total of *k* = 10 experiments were extracted from the included studies and can be viewed in Appendix H of the *Supplementary Materials*. Seven studies were MDD^29,31,41–45^, and one was BP^41^. A cluster-level FWE correction threshold of *p* <.05 was used, with an uncorrected value of *p* < .001. A rigorous threshold of 5,000 permutations was applied. The ALE meta-analysis reported significantly more convergence of activation (patients > controls) in ten cluster regions with ten peak coordinates. The first peak coordinate (*x* = −22, *y* = 50, *z* = 12) was reported in the left medial frontal gyrus (BA9), spreading to the left SFG (BA10), and left MFG (BA10). Peak coordinates were reported in the right orbital gyrus (*x* = 20, *y* = 20, *z* = −32) (BA47, BA11) and in the left (*x* = −54, *y* = 22, *z* = 10) inferior frontal gyrus (IFG) (BA44, BA45). Peak coordinates (*x* = −46, *y* = 40, *z* = −8) were reported in the left MFG (BA47) and in the right and left (*x* = 4, *y* = −2, *z* = 52) medial frontal gyrus (BA6). Peak coordinates (*x* = 0, *y* = 26, *z* = 16) were reported in the left anterior cingulate gyrus (BA32), spreading to the left cingulate gyrus (BA24, BA33). A peak coordinate (*x* = 42, *y* = 2, *z* = 14) was reported in the right insula (BA13) and in the left lentiform nucleus (putamen) (*x* = −26, *y* = −6, *z* = 6). Finally, peak coordinates were reported in left claustrum (*x* = −38, *y* = −14, *z* = −4) and the right claustrum (*x* = 42, *y* = −14, *z* = −8), spreading to the insula (BA13). All locations were reported to be 100% grey matter. The result is available in Table 1. And can be viewed in Fig. 4.

### Subgroup analysis for anxiety disorders during implicit emotion regulation

Parameters were set to a cluster-level FWE correction threshold of *p* <. 05, with an uncorrected value of *p* < .001. A rigorous threshold of 5,000 permutations was applied. However, no significant clusters were reported. For further exploration, the threshold for analysis was reduced to 1,000 permutations. Yet, no significant clusters were reported. An interpretation of this result is presented in the Discussion. Extracted experiments for both activation contrasts and can be viewed in Appendix I of the *Supplementary Materials*.

## Discussion

Overall, whole-brain ALE meta-analysis reported convergence of hypoactivation during implicit emotion regulation in mood and anxiety disorder patients in two cluster regions. The first region was the right medial frontal gyrus (BA9), which extended over the right anterior cingulate gyrus (BA32). The second cluster region was the left MTG (BA21), extending over the left STG (BA22). In contrast, ALE meta-analysis reported convergence of hyperactivation in mood and anxiety disorder patients in one cluster region. This region was the left medial frontal gyrus (BA9, BA10), spreading to the left MFG and left SFG.

The medial frontal gyrus converges ventral and dorsal attention networks, which involves the mediation of top-down control over emotions, automatic behaviours, impulsivity, and mental states^56–61^. The anterior cingulate gyrus integrates amygdala and PFC activity regulating emotions, attention, inhibition, uncertainty, reward, and impulsivity^62^. Collectively, the medial frontal gyrus and the anterior cingulate gyrus form part of the medial prefrontal cortex (mPFC), which is responsible for learning associations between events and their corresponding adaptive emotional response^63^. To achieve this, the mPFC converges information from subcortical regions and other PFC regions^62,64^, regulating top-down control of limbic responses towards sensory information; including fear conditioning, attention, emotion regulation, motivation, decision-making, uncertainty, reward, and memory^62–67^. Previous research has outlined recruitment of the mPFC during implicit emotion regulation in healthy populations^12^. The results of the present ALE meta-analysis suggest the mPFC is an area of dysfunctional activation in mood and anxiety disorder patients during implicit emotion regulation. Specifically, the present ALE meta-analysis reported convergence of attenuation in patients in the right mPFC (including the ACC) during implicit emotion regulation. Substantial evidence suggests PFC attenuation in patients is a result of an inability to effectively regulate limbic responses due to weak fronto-limbic connectivity^30^. This has been noted in the ventral and dorsal medial PFC, including the ACC^30,68,69^, areas outlined in the present review’s findings. Such observations have also been observed in previous studies of mood and anxiety disorder patients during implicit emotion regulation^20–23,25,27,33,34,37,38^ and is further supported by general amygdala-PFC fear circuitry models and models of emotional dysregulation^19,28^. The present ALE meta-analysis simultaneously reported convergence of hyperactivation in patients in the left mPFC, which could highlight an adaptive compensatory mechanism. However, further investigation is required to explore this claim. Increased activation in the left mPFC may also be representitive of dysfunctional cognitive-emotional processes such as rumination and worry, as the left hemisphere is typically involved in language processing^70,71^. This may further be supported by the present findings of the ALE meta-analysis that reported hypoactivation in the left STG, which extended over the MTG; areas involved in language processing, mental states, social perception and memory^56,72,73^. A previous meta-analysis has also highlighted dysfunction in these regions during explicit emotion regulation in depressed patients^13^. Given such areas are highlighted in thought-related disorders, this finding may also be linked to the maladaptive nature of rumination and worry^72,74^. In summary, given the review’s findings and that patients experience difficulties across the functions of the mPFC, this area is highlighted as a ROI for future investigation. A recommendation for future research would be to investigate the nuanced lateralised differences of mPFC activation during implicit emotion regulation in patients.

While the lateral PFC has previously been outlined during implicit emotion regulation in patients^19,22,24^, this was not highlighted in the present review’s findings. One explanation for this may be that the lateral PFC is mainly involved during emotion regulation that requires higher-order cognitive processes or explicit emotion regulation^12^. Furthermore, the lateral PFC recruits the mPFC (including the ACC) during the suppression of limbic reactivity^62^. Therefore, the mPFC may act as a mediator between activation in the lateral PFC and the amygdala, responding quicker than the lateral regions during implicit emotion regulation or possibly overruling lateral PFC processing. In fact, while the lateral PFC is not present in all mammals, the mPFC exists in all mammals and may be an example of the primitive circuitry preserved in humans which supports higher-order cognitive functions that are unique to humans^62^.

The amygdala was not reported in the findings of the present ALE meta-analysis. This is surprising given the amygdalae consolidate the processing of emotion, motivation, and behaviour, and play a crucial role in emotional reactivity, autonomic responses, mental states, memory, and in the pairing of an emotional response from a stimulus to its emotional value or fear conditioning^12,68,75–81^. Several previous studies have outlined the amygdala during implicit emotion regulation in patients^19,24,29–34^. However, the present non-significant finding may collectively reflect the cognitive processes of implicit emotion regulation rather than emotional reactivity or fear conditioning^69,79–81^. Further research is needed to clarify the exact function of the amygdala during implicit emotion regulation. Research may investigate the several subdivisions of the amygdala which are known to regulate various aspects of behaviour^77,81,82^.

Although additional experiments are needed to confidently interpret the findings of the separate subgroup analyses for mood and anxiety disorders^83^, analyses were performed for exploratory purposes. Regarding mood disorders, findings of both hypoactivation and hyperactivation in patients reflected the overall outcomes, with additional areas identified. With regards to convergence of hypoactivation in mood disorder patients, the parahippocampul gyrus (BA36, BA35) and culmen were highlighted. The parahippocampul gyrus is linked to episodic memory, working memory, and temporal memory^84^. Given that mood disorders are also associated with memory problems, it is certainly plausible that related memory regions are also affected during implicit emotion regulation. The culmen forms part of the cerebellum and while not traditionally linked to emotion processing, recent research suggests a role of the cerebellum in emotional processing, emotional learning, and emotional control^85^, perhaps due to its connections to the PFC and limbic system. Further research is needed to investigate this with respect to implicit emotion regulation. On the contrary, convergence of hyperactivation in mood disorder patients was observed in several regions including the orbital gyrus (BA47, BA11), IFG (BA44, BA45), insula (BA13), lentiform nucleus, and claustrum. Previous research had highlighted dysfunctional activation during implicit emotion regulation in patients in the OFC^25,26^ and insula^19,23,28^. The insula is known to be implicated in sensory processing, valence processing, emotion processing, reward, interoception, and decision-making^86^. Furthermore, the insula converges sensory, limbic, and PFC regions, including connections to the mPFC and ACC^86^, areas outlined in this review. Therefore, it is plausible that the insula may be dysfunctional during implicit emotion regulation in patients. Further investigation is needed. Interestingly, the claustrum was highlighted in this review, and while the function of the claustrum remains largely unknown^87^, it has been linked to consciousness, attention, cognitive control, and salience processing^87,88^. Given this, the findings of the present ALE meta-analysis, and that the claustrum has extensive connections to the whole cerebral cortex^87,88^, the claustrum may in fact be recruited during implicit emotion regulation. Hyperactivity of the claustrum in patients may be representative of impaired cognitive control while implicitly regulating emotions or may represent increased neural effort to sustain implicit emotion regulation abilities. Importantly, further investigation is needed to outline the claustrum’s role during implicit emotion regulation.

Regarding anxiety disorders, no significant clusters were reported in this model. This may be due to heterogeneity between anxiety disorders. For instance, neural activity specific to GAD patients has been observed in response to threat in several neural regions compared to SAD and PD patients^23^. However, it is important to note that there were an insufficient number of studies for adequate power in the anxiety disorder subgroup^83^, which may account for this non-significant result. Further studies are needed to confidently perform ALE meta-analysis for the anxiety disorders subgroup in order to draw meaning interpretations.

### Clinical implications

In addition to traditional diagnostic methods that classify symptoms across populations to establish a diagnosis (i.e., DSM-5), the RDoC framework aims to enhance the assessment, diagnosis, and treatment of psychiatric disorders by investigating the many domains of dysfunction within patients. To achieve this, the RDoC framework integrates findings from neurosciences, genetics, genomics, and behavioural sciences^36^. The results of the present fMRI meta-analysis reported mPFC dysfunction in patients during implicit emotion regulation, which is suggested to be a transdiagnostic characteristic of mood and anxiety disorders. Therefore, understanding the neural circuitry of the mPFC during implicit emotion regulation may be critical in improving our knowledge of the pathophysiology of mood and anxiety disorders. Such research may provide insights into their future assessment, diagnosis, and treatment. In addition, the right hemisphere appears to be attenuated in patients during implicit emotion regulation, while the left hemisphere shows hyperactivity. This may potentially be compensatory activation which may provide important information on the underlying mechanisms of implicit emotion regulation in patients. It would be pertinent to evaluate how mPFC activity alters over the course of treatment interventions, including both psychopharmacological and cognitive behavioural interventions. In fact, a meta-analysis reported that conscious reappraisal is associated with activation in the mPFC and ACC^89^. This could also provide information on the transdiagnostic nature of implicit emotion regulation. However, further investigation is required. The current findings offer support to the NIHM framework of developing diagnostic systems that are more predictive of treatment outcomes. Integrating findings from psychiatry and clinical neurosciences will help uncover more about these findings and how they translate to the clinic.

A previous meta-analysis of depressed participants reported dysregulation of the MTG, STG, and parahippocampal gyrus during explicit emotion regulation^13^. These areas were also highlighted in the present review and warrants further investigation. Along with the main findings of the present ALE meta-analysis, reporting dysregulation of the mPFC in patients during implicit emotion regulation, it appears that dysregulation of the lateral PFC may be related to maladaptive explicit emotion regulation and dysfunction of the medial PFC may be related to maladaptive implicit emotion regulation. This is supported by another meta-analysis that has highlighted neural differences during explicit vs. implicit emotion processing^90^. Therefore, this is a crucial area of investigation for future research.

Finally, the findings have implications for the effectiveness of explicit emotion regulation strategies. For instance, dysfunctional implicit emotion regulation may affect explicit emotion regulation through automatic spontaneous attention shifts^11^. Interventions that focus on conscious strategies such as cognitive behavioural therapy or rational emotive behaviour therapy may be constrained by maladaptive implicit emotion processing tendencies. Therefore, it would be important to investigate how dysfunctional implicit emotion regulation affects the success of conscious emotion regulation strategies and interventions. Further investigation is necessary.

### Limitations and future directions

The review focused on mood and anxiety disorders due to their shared aetiology and frequent co-morbidity^42,91^. However, neural differences have been observed between conditions. For instance, MDD patients have exhibited reduced activation in the anterior cingulate gyrus compared with bipolar patients during implicit emotion regulation^37^. While GAD-specific activity in response to threat has been observed in several neural regions compared to SAD and PD patients^23^. Future ALE meta-analyses would examine psychiatric conditions separately. Additionally, the severity of a disorder, along with whether the disorder was a first episode or a relapsing disorder was not investigated. Future reviews would consider this. Future reviews would also focus on the inclusion of psychotropic medication and/or active psychotherapy. It may be hypothesised that appropriate intervention stabilises neural dysfunction during implicit emotion regulation. Finally, future meta-analyses may extend focus to other conditions excluded here, such as psychotic disorders or personality disorders, which may provide additional information on the transdiagnostic status of implicit emotion regulation.

The review aimed to investigate implicit emotion regulation across emotion processing in general as opposed to specific categories of emotions. However, neural differences have been reported in response to different emotions^13^. For instance, during implicit emotion regulation, generalised social phobic patients have exhibited increased amygdala activation in response to angry and contemptuous facial stimuli compared to happy facial stimuli^92^. Future reviews would examine emotions separately which would highlight important implicit emotion processing differences in patients, or may approach the research question from another perspective such as dimensional emotion systems, alternative to basic emotions.

Finally, research should explore the DMN and SN with respect to implicit emotion regulation in patients. In healthy individuals, the DMN is a large network of regions active when the mind is at rest and typically supressed when engaging in behaviours^14^. However, PTSD patients have exhibited increased activity during implicit emotion regulation^19^, while altered DMN activity has been linked to rumination in MDD^15^. The SN is responsible for the flexible shifting of attention between internal and external tasks. Dysfunctional activation of the SN has also been linked to mood and anxiety disorders^16^, which may account for difficulties of disengaging from negative information such as rumination and worry. Furthermore, the mPFC and ACC are largely implicated in the DMN and SN, respectively. As these neural regions were outlined in this review, further investigation would improve our understanding of implicit emotion regulation in patients and may lead to improved outcomes of assessment, diagnosis, and treatment.

## Conclusion

This review outlines several areas of convergence of both hypoactivation and hyperactivation in mood and anxiety disorder patients during implicit emotion regulation. The overall findings indicate attenuation in the right mPFC and hyperactivation in the left mPFC in patients during implicit emotion regulation. The findings offer important information on the pathophysiology, diagnosis, and treatment of mood and anxiety disorders.

## Methods

This meta-analysis was registered with PROSPERO, the International Prospective Register of Systematic Reviews (Registration ID: CRD42022360082). The review followed both neuroimaging meta-analysis protocol^83^ and the Preferred Reporting Items for Systematic Reviews and Meta-Analyses (PRISMA)^40^.

### Eligibility criteria

The present fMRI review focused on mood and anxiety disorders due to their common co-morbidity and shared aetiology^42,91^. Patients were recruited from several demographics including hospitals (mainly outpatients), clinics, and community residents. Patients were only included when a psychiatric diagnosis was based on established diagnostic measurements such as the Structured Clinical Interview for the Diagnostic and Statistical Manual of Mental Disorders Fifth Edition (DSM-5)^93^ or the International Classification of Disease^94^. This excluded self-report cutoff measures as a means of a valid psychiatric diagnosis. Included study designs comprised of blinded and unblinded randomised clinical controlled comparison studies and experimental studies. Observational studies and case studies were excluded as they do not typically have a control condition. Healthy control samples acted as the controls in this review and were reported to have no history of psychiatric disorders, which were verified by structured clinical interviews. Functional MRI studies were the focus of this review and studies were included where authors reported whole-brain foci for paradigm-based measures of implicit emotion regulation, contrasted between patients and healthy controls. Thus, ROI studies were excluded. In this review, implicit emotion regulation was defined where participants automatically modulated their emotions without an active strategy. We included tasks that investigated attempts at regulating emotional responses, as opposed to tasks that observe neural responses towards emotional stimuli, which may involve responding to emotions without regulating them. Studies were excluded that investigated explicit forms of emotion regulation (i.e., conscious reappraisal) and studies that did not utilise a behavioural performance task.

Due to inconsistent findings in the fMRI literature, strict exclusion criteria were outlined. While patients with mood and/or anxiety disorder comorbidities were included, patients with a history of psychosis, a neurological condition, personality disorder, suicidal ideation, or psychiatric hospitalisation were excluded. With respect to bipolar disorder where psychosis has a higher prevalence, patients were only included where authors had clearly stated psychosis was not present in their sample. Studies where patients were in remission were excluded due to possible confounding effects. Additionally, studies were excluded if most of the patient sample were affected by psychotropic medications (i.e., SNRIs, SSRIs, or benzodiazepines) on the day of testing, and if they were receiving ongoing psychotherapy. Finally, heart disease and hypertension were excluded due to possible associations with neurodegenerative diseases. Individuals with MRI contraindications were excluded.

### Search strategy

Peer-reviewed articles were screened from inception up until the end of April 2024. Searches were performed in databases which included Web of Science, Scopus, PubMed/MEDLINE, and BrainMap. Reference lists were also screened for relevant articles. Due to file-drawer concerns in line with clinical research, grey literature was searched in the OpenGrey database. Titles, abstracts, and keywords were screened using a total of 53 search terms relating to implicit emotion regulation in mood and anxiety disorders. Search terms were truncated and combined with Boolean operators and MeSH searching. An example of search terms included were: “implicit OR automatic OR unconscious OR nonconscious” AND “emotion* regulat*” OR “affect* regulat*” OR “mood regulat*” AND “mood disorder*” OR depress* OR “major depress* disorder” OR unipolar”. Although PTSD and OCD are no longer classified as anxiety disorders in the DSM-5, they were included in the searches due their substantial presentation of anxiety symptoms. Articles sought after were written in English Language due to limited resources for translating articles from other languages. The full search strategy can be found in Appendix D of *Supplementary Materials.*

### Study selection and data extraction

Data extracted included author(s) and year of publication, sample size, psychiatric condition, diagnostic measurement, comorbidities, and study characteristics. To carry out a coordinate-based meta-analysis, whole-brain neural foci (*x*,* y*,* z*) were extracted for paradigm-based measures of implicit emotion regulation and contrasted as either patients (major depressive disorder/unipolar depression, bipolar disorder, generalised anxiety disorder, generalised social phobia/social anxiety disorder, panic disorder, and post-traumatic stress disorder) or healthy controls. Neural foci were extracted as reported by authors. In instances where authors did not provide data for both activation contrasts, available data was extracted. Acquisition space was also extracted. On occasions where a single study included more than one psychiatric condition (i.e., a separate sample of MDD and GAD patients), data were extracted as separate samples. To manage studies with multiple experiments, activation contrasts were only selected if they met the review’s aims. Further following the reviews aims, data was extracted and combined to create an ‘emotion’ variable as opposed to evaluating specific dimensions of emotion processing. Neutral stimulus conditions were omitted from extraction. Article suitability, full-text screening, and data extraction were conducted independently by two blinded reviewers (S.D.P.D and H.C) and disagreements were resolved by a third reviewer (S.C).

### Quality assessment

The Newcastle-Ottawa Scale adapted for cross-sectional studies^55^ was modified for the purpose of this review. The quality of each study was assessed on whether patients were typical of the general population (e.g., severity and comorbidities) and whether healthy controls were comparable (e.g., age, gender or IQ.) Studies were additionally evaluated on whether patients had a verified mental health diagnosis (e.g., DSM-5 by a qualified person) and whether a structured clinical interview was carried out in both patients and healthy controls to confirm or reject diagnosis. Finally, samples were assessed on whether a diagnosis was performed by external persons (i.e., within an outpatient clinic) or by the research team.

### Activation likelihood estimation meta-analysis

A meta-analysis of neural correlates was performed using an ALE meta-analysis. ALE meta-analysis determines above-chance convergence of foci between experiments^95^, or the convergence of differences in neural activation between patients and healthy controls during implicit emotion regulation. To achieve this, the ALE meta-analysis models coordinate locations (*x*,* y*,* z*) as a 3D Gaussian distribution of probability (i.e., empirical model of spatial uncertainty) or the likelihood of activation during implicit emotion regulation between patients and controls. Thus, replacing foci with a probability distribution and computing an ALE score^95^. Sample sizes were entered using the lowest sample size between patient and control groups, reducing computing bias. A minimum cluster level of 200mm^3^ was selected for cortical neural regions. For subcortical regions, the minimum cluster size threshold was removed in line with recent studies that have documented subcortical structures smaller than 50mm³ in MDD patients^96^. A FWE correction threshold of *p* < .05 was used, with an uncorrected value of *p* < .001. A strict threshold of 5,000 permutations was selected. If results yielded non-significant results, the threshold of analysis was reduced to 1,000 permutations for exploratory purposes. Only peak coordinates were reported. Overall, two ALE meta-analyses of convergence for whole-brain foci were performed contrasted by convergence of increased activation in patients (patients > controls) and convergence of decreased activation in patients (controls > patients). Four subsequent subgroup ALE meta-analyses were performed for exploratory purposes, which comprised of convergence of increased activation in mood disorder patients (patients > controls), convergence of decreased activation in mood disorder patients (controls > patients), convergence of increased activation in anxiety disorder patients (patients > controls), and convergence of decreased activation in anxiety disorder patients (controls > patients). The ALE meta-analysis was performed with GingerALE version 3.0.2., which generated anatomical labelling of clusters. All coordinates were reported in Montreal Neurological Institute (MNI) space. Studies that reported Talairach coordinates were converted to MNI space using BrainMap (GingerALE). The results were added as an overlay onto a MNI152 template in MRIcroGL. All figures were created in MRIcroGL.

## Electronic supplementary material

Below is the link to the electronic supplementary material.

Supplementary Material 1

## Data Availability

Raw and generated data, as well as data analysed during this review, are available within this published article and its supplementary materials.
