# Supplementary material for: Neural correlates of implicit emotion regulation in mood and anxiety disorders: an fMRI meta-analytic review
Source: Sci Rep. 2025 Jun 4;15:19564. doi: 10.1038/s41598-025-03828-5 (PMC12137648; doi:10.1038/s41598-025-03828-5)
Supplement: Supplementary file 1 — Supplementary Material 1 [file 41598_2025_3828_MOESM1_ESM.pdf]

SUPPLEMENTARY MATERIALS

Neural Correlates of Implicit Emotion Regulation in Mood and Anxiety Disorders: An fMRI Meta-Analytic Review

Stefan Daniel Paul Dalton, Holly Cooper, Ben Jennings, Survjit Cheeta

Appendix A: PRISMA checklist

Section and Topic	Item #	Checklist item	Location where item is reported
**TITLE**			
Title	1	Identify the report as a systematic review.	p. 1
**ABSTRACT**			
Abstract	2	See the PRISMA 2020 for Abstracts checklist.	p. 2
**INTRODUCTION**			
Rationale	3	Describe the rationale for the review in the context of existing knowledge.	pp.3-4
Objectives	4	Provide an explicit statement of the objective(s) or question(s) the review addresses.	pp.4-5
**METHODS**			
Eligibility criteria	5	Specify the inclusion and exclusion criteria for the review and how studies were grouped for the syntheses.	pp. 32-35
Information sources	6	Specify all databases, registers, websites, organisations, reference lists and other sources searched or consulted to identify studies. Specify the date when each source was last searched or consulted.	p. 33 & Figure 1
Search strategy	7	Present the full search strategies for all databases, registers and websites, including any filters and limits used.	Supplementary Materials
Selection process	8	Specify the methods used to decide whether a study met the inclusion criteria of the review, including how many reviewers screened each record and each report retrieved, whether they worked independently, and if applicable, details of automation tools used in the process.	pp. 32-34
Data collection process	9	Specify the methods used to collect data from reports, including how many reviewers collected data from each report, whether they worked independently, any processes for obtaining or confirming data from study investigators, and if applicable, details of automation tools used in the process.	pp. 32-34
Data items	10a	List and define all outcomes for which data were sought. Specify whether all results that were compatible with each outcome domain in each study were sought (e.g. for all measures, time points, analyses), and if not, the methods used to decide which results to collect.	p. 34
	10b	List and define all other variables for which data were sought (e.g. participant and intervention characteristics, funding sources). Describe any assumptions made about any missing or unclear information.	p. 34
Study risk of bias assessment	11	Specify the methods used to assess risk of bias in the included studies, including details of the tool(s) used, how many reviewers assessed each study and whether they worked independently, and if applicable, details of automation tools used in the process.	p. 34 & Table 2
Effect measures	12	Specify for each outcome the effect measure(s) (e.g. risk ratio, mean difference) used in the synthesis or presentation of results.	p. 34
Synthesis methods	13a	Describe the processes used to decide which studies were eligible for each synthesis (e.g. tabulating the study intervention characteristics and comparing against the planned groups for each synthesis (item #5)).	p. 33 & Table 1
	13b	Describe any methods required to prepare the data for presentation or synthesis, such as handling of missing summary statistics, or data conversions.	p. 34

Section and Topic	Item #	Checklist item	Location where item is reported
	13c	Describe any methods used to tabulate or visually display results of individual studies and syntheses.	p. 34 & Table 1
	13d	Describe any methods used to synthesize results and provide a rationale for the choice(s). If meta-analysis was performed, describe the model(s), method(s) to identify the presence and extent of statistical heterogeneity, and software package(s) used.	p. 34
	13e	Describe any methods used to explore possible causes of heterogeneity among study results (e.g. subgroup analysis, meta-regression).	p. 34
	13f	Describe any sensitivity analyses conducted to assess robustness of the synthesized results.	p. 34
Reporting bias assessment	14	Describe any methods used to assess risk of bias due to missing results in a synthesis (arising from reporting biases).	p. 33
Certainty assessment	15	Describe any methods used to assess certainty (or confidence) in the body of evidence for an outcome.	p. 33
**RESULTS**			
Study selection	16a	Describe the results of the search and selection process, from the number of records identified in the search to the number of studies included in the review, ideally using a flow diagram.	p. 6 & Figure 1
	16b	Cite studies that might appear to meet the inclusion criteria, but which were excluded, and explain why they were excluded.	p. 6 & Figure 1
Study characteristics	17	Cite each included study and present its characteristics.	p. 8 & Table 1
Risk of bias in studies	18	Present assessments of risk of bias for each included study.	p. 13
Results of individual studies	19	For all outcomes, present, for each study: (a) summary statistics for each group (where appropriate) and (b) an effect estimate and its precision (e.g. confidence/credible interval), ideally using structured tables or plots.	Table 3 & pp.17-23
Results of syntheses	20a	For each synthesis, briefly summarise the characteristics and risk of bias among contributing studies.	pp.17-23
	20b	Present results of all statistical syntheses conducted. If meta-analysis was done, present for each the summary estimate and its precision (e.g. confidence/credible interval) and measures of statistical heterogeneity. If comparing groups, describe the direction of the effect.	pp.15-23
	20c	Present results of all investigations of possible causes of heterogeneity among study results.	Table 1
	20d	Present results of all sensitivity analyses conducted to assess the robustness of the synthesized results.	Figures 2, 3 & 4
Reporting biases	21	Present assessments of risk of bias due to missing results (arising from reporting biases) for each synthesis assessed.	Table 2
Certainty of evidence	22	Present assessments of certainty (or confidence) in the body of evidence for each outcome assessed.	Table 2
**DISCUSSION**			
Discussion	23a	Provide a general interpretation of the results in the context of other evidence.	pp. 24-27
	23b	Discuss any limitations of the evidence included in the review.	pp. 24-27
	23c	Discuss any limitations of the review processes used.	pp. 29-30
	23d	Discuss implications of the results for practice, policy, and future research.	pp. 27-28
**OTHER INFORMATION**			
Registration and	24a	Provide registration information for the review, including register name and registration number, or state that the review was not registered.	p. 31

Section and Topic	Item #	Checklist item	Location where item is reported
protocol	24b	Indicate where the review protocol can be accessed, or state that a protocol was not prepared.	p. 31
	24c	Describe and explain any amendments to information provided at registration or in the protocol.	n/a
Support	25	Describe sources of financial or non-financial support for the review, and the role of the funders or sponsors in the review.	p. 44
Competing interests	26	Declare any competing interests of review authors.	p. 44
Availability of data, code and other materials	27	Report which of the following are publicly available and where they can be found: template data collection forms; data extracted from included studies; data used for all analyses; analytic code; any other materials used in the review.	See: Supplementary Materials

From: Page MJ, McKenzie JE, Bossuyt PM, Boutron I, Hoffmann TC, Mulrow CD, et al. The PRISMA 2020 statement: an updated guideline for reporting systematic reviews. BMJ 2021;372:n71. doi: 10.1136/bmj.n71.
This work is licensed under CC BY 4.0. To view a copy of this license, visit https://creativecommons.org/licenses/by/4.0/

Appendix B: Newcastle-Ottawa Scale Adapted for Cross-Sectional Studies

This scale has been adapted from the Newcastle-Ottawa Quality Assessment Scale to provide quality assessment of cross-sectional studies included in the review: *Neural Correlates of Implicit Emotion Regulation in Mood and Anxiety Disorders: An fMRI Meta-Analytic Review*

Selection: (Maximum 5 stars)

1. Representativeness of the clinical sample:

    a) Truly representative of the average patient in the community with mental illness. (E.g., diagnosis, severity, and comorbidities.) *
    b) Somewhat representative of the average patient in the community with mental illness. (E.g., diagnosis, severity, and comorbidities.) *
    c) Selected group of users. (Convenience sampling.)
    d) No description of the included sample.

2. Selection of the non-clinical sample:

    a) Drawn from comparable sample. (E.g., age, gender.) *
    b) Drawn from an alternative sample.
    c) No information provided.

3. Ascertainment of exposure:

    a) Verified mental health diagnosis (E.g., DSM-5 or ICD-10 by qualified persons i.e., physician or psychiatrist, GP records, hospital records.) *
    b) Structured clinical interview. *
    c) Self-report.
    d) No information provided.

4. Demonstration that outcome of interest was not present in non-clinical sample:

    a) Non-clinical sample did not meet diagnostic criteria for mental health diagnosis (E.g., DSM-5 or ICD-10.) *
    b) Self-report.
    c) No information provided.

Comparability: (Maximum 2 stars)

5. Comparability of samples in the different outcome groups based on design or analysis.

    a. Data and/or results corrected for relevant outcomes and confounding variables controlled (e.g. age, sex, IQ etc.), or other. **
    b. Data and/or results not corrected for relevant outcomes and confounding variables not controlled, other, or information not provided.

Outcome: (Maximum 3 stars)

6. Assessment of outcome:

a) Independent or blinded diagnosis using verified measures. (E.g., DSM-5 or ICD-10.) **
b) Unblinded diagnosis using verified measures. (E.g., DSM-5 or ICD-10.) *
c) Self-report measures.
d) No description.

7. Statistical test:

a) Statistical test used to analyse the data is clearly described, is appropriate, and describes the measurement of the association. (E.g., p value). *
b) Statistical test is not appropriate or not described.

Cross-sectional Studies:

Excellent: 9-10 points

Good: 7-8 points

Satisfactory: 5-6 points

Unsatisfactory: 0 to 4 points

Appendix C: Table 3 Corresponding Studies

[1]	Wang et al., 2021
[2]	Thomaes et al., 2012
[3]	Arnone et al., 2012
[4]	Blair et al., 2011
[5]	Blair et al., 2012
[6]	Schwarzmeier et al., 2019
[7]	Mazza et al., 2012
[8]	Korgaonkar et al., 2021
[9]	Chechko et al., 2013
[10]	Bürger et al., 2017
[11]	Gaebler et al., 2013
[12]	Etkin & Schatzberg, 2011
[13]	Feldker et al., 2018
[14]	Yu et al., 2015
[15]	Neumeister et al., 2018
[16]	Cerullo et al., 2014
[17]	Klumpp et al., 2013
[18]	Kaldewaij et al., 2019
[19]	Kraus et al., 2018
[20]	Palm et al., 2011
[21]	Mitterschiffthaler et al., 2008
[22]	Ruhé et al., 2011
[23]	Fodl et al., 2009
[24]	Heitmann et al., 2017

Appendix D: Search Strategy

**WoS/ Scopus/ Pubmed/MEDLINE**
implicit OR automatic OR unconscious OR nonconscious OR uncontrolled OR effortless AND “emotion* regulat*” OR “affect* regulat*” OR “mood regulat*” OR “stress regulat*” OR strateg* AND psychopathology OR disorder* OR “mood disorder*” OR depress* OR “major depress* disorder” OR unipolar OR bipolar OR anxiety OR “anxiety-type” OR “generalised anxiety disorder” OR “generalized anxiety disorder” OR “panic disorder” OR “social anxiety” OR “health anxiety” OR phobia* OR “obsessive compulsive disorder” AND “affect labelling” OR reappraisal OR suppression OR extinction OR spontaneous OR rumination OR distraction OR attention* OR avoidance OR “emotion* conflict” OR “error related” OR mindfulness OR acceptance OR “go nogo” OR “problem solving” OR “reinforcer revaluation” OR modification OR habit* OR priming OR “emotion* Stroop” OR distancing OR concentration OR “goal pursuit” OR latency OR “reversal learning” OR “emotion* reactivity”
**BrainMap/ Sleuth**
Subjects/ Diagnosis/ anxiety, depression, generalized anxiety disorder, major depressive disorder, obsessive compulsive disorder, panic disorder, PTSD, social anxiety disorder, specific phobia, unipolar disorder, bipolar disorder AND Experiments/ Behavioural Domain/ Emotion/ All Subtypes AND Experiments/ Paradigm classes/ emotion induction/ affective pictures, affective words, Stroop-Emotional, Go/ No-Go

Appendix E: Mood and Anxiety Disorders Foci (controls>patients)

// Reference=MNI

// Cerullo M A, 2016

-39.39 57.77 -32.44

18.36 108.01 -7.2

-38.7 -4.22 32.97

-55.13 -5.25 -20.28

-16.7 -2.11 -24.33

32.07 8.55 67.89

-13.65 25.95 -33.41

2.8 37.01 25.69

// Cerullo M A, 2016

-42.62 64.1 -32.93

-64.71 -1.63 -13.78

-26.17 -33.63 -18.23

-29.08 -60.14 10.79

-58.51 -.47 -40.72

// Cerullo M A, 2016

-39.39 57.77 -32.44

18.36 108.01 -7.2

-38.7 -4.22 32.97

-55.13 -5.25 -20.28

-16.7 -2.11 -24.33

-42.62 64.1 -32.93

-64.71 -1.63 -13.78

-26.17 -33.63 -18.23

32.07 8.55 67.89

-13.65 25.95 -33.41

2.8 37.01 25.69

-29.08 -60.14 10.79

-58.51 -.47 -40.72

// Arnone D, 2012

28	-88	25
-11	-81	5
32	-70	-10
14	-88	-5
-25	-84	30
32	-88	10
-42	7	45
39	-4	55
56	-32	-10
53	-70	0
35	-7	60
-11	-74	45

// Blair K S, 2011

12.17 -98.96 7.44

// Kraus J, 2018

32 -2 -14

// Schwarzmeier H, 2019

24	29	37
42	-25	22
-42	-19	43
-60	-19	22
-54	-4	13
-57	-37	46
39	-4	40
-54	-34	-14
-3	29	61

-6 -52 34

-15 2 34

0 20 64

6 -73 43

45 -43 25

54 -43 31

39 11 37

// Mazza M, 2012

-11.46 -99.25 -7.88

// Wang, 2021

23.77 -45.85 73.5

13.65 36.45 58.81

11.39 27 49.7

// Korgaonkar, 2021

-18 -26 26

70 -52 36

-28 -58 -42

-12 -72 -46

10 -70 -48

-10 30 12

-30 -4 16

28 -68 -8

24 -56 -44

-20 -48 -26

-40 12 12

-24 30 28

-30 -52 -10

-18 4 36

-16 -60 48

-16	26	34
-24	40	-10

// Blair, 2012

-5	26	24
57	-33	21
-63	-42	31
-31	-58	44

// Blair, 2012

20	-7	-18

// Etkin, 2011

-10	28	-2
-4	40	-16

// Etkin, 2011

18	2	-16

// Yu, 2015

50	20	40

// Burger, 2017

−24	4	4
−52	−6	−4
2	46	26
−22	50	10
4	40	28
24	54	0
−20	42	24
4	48	26
4	40	28

26 54 -4

// Chechko, 2013

34 −54 −28

−48 18 22

50 28 14

46 −66 −12

10 −80 −22

−8 −84 −28

−2 6 70

40 −48 46

−28 −66 44

54 26 −4

48 -64 -14

-60 -4 -10

-60 -4 -10

// Palm M E, 2011

33.14 29.9 -23.8

-47.83 34.39 -17.26

-8.96 46.43 -16.87

16.91 65.77 -18.11

-15.43 69.47 -11.2

-31.37 52.79 6.41

17.32 63.36 13.5

39.73 31.82 -15.13

-50.72 31.55 9.96

4.29 49.72 6.11

// Ruhe H G, 2012

50 20 -6

-34 22 -6

42	16	27
48	-2	51
-54	16	0
-4	10	63
-42	-54	-21
-8	26	42
-6	-20	51
-4	10	63
50	20	-6
-34	22	-6
42	16	27
-42	-54	-21
46	-40	-24
-16	-38	-21
58	-6	-12
-12	-22	48
54	32	3
44	2	48
54	-48	12
48	-36	6
-40	-54	-18

Appendix F: Mood and Anxiety Disorders Foci (patients>controls)

// Reference=MNI

// Cerullo M A, 2016

70.06 13.67 6.81

-23 51.69 11.38

-7.29 38.67 -34.55

-54.98 22.69 10.83

6.01 76.64 42.41

-32.09 -54.94 37.15

-45.56 40.5 -7.5

2.59 100.71 23.7

21.65 23.24 -30.24

41.3 1.32 14.86

-26.07 -6.2 6.23

// Cerullo M A, 2016

63.58 9.69 -2.82

-38.95 -13.39 -3.06

-.44 26.71 16.56

24.61 103.08 -30.28

18.43 19.79 -33.26

-45.56 89.88 11.78

3.13 -2.43 52.42

-26.57 51.25 -35.38

41.18 -13.17 -7.34

35.14 43.46 64.93

// Cerullo M A, 2016

70.06 13.67 6.81

-23 51.69 11.38

-7.29 38.67 -34.55

-54.98 22.69 10.83

6.01	76.64	42.41
-32.09	-54.94	37.15
-45.56	40.5	-7.5
2.59	100.71	23.7
21.65	23.24	-30.24
41.3	1.32	14.86
-26.07	-6.2	6.23
63.58	9.69	-2.82
-38.95	-13.39	-3.06
-.44	26.71	16.56
24.61	103.08	-30.28
18.43	19.79	-33.26
-45.56	89.88	11.78
3.13	-2.43	52.42
-26.57	51.25	-35.38
41.18	-13.17	-7.34
35.14	43.46	64.93

// Arnone D, 2012

56	-7	30
-14	-53	50
18	-56	-30

// Feldker K, 2017

2.28	-40.24	-2.95
19.48	-43.51	8.28
-9.55	-31.22	-9.15
27.02	-54.92	11.38
-49.2	9.07	-7.61
-39.58	-.73	-11.36
-64.02	7.18	10.56
-36.4	-8.63	-17.42

-50.09 5.49 15.01

// Kaldewaij R, 2019

6 54 6

6 62 14

38 -86 14

-40 -86 -20

-6 -56 18

// Blair K S, 2011

8.33 47.22 -14.82

-28.98 -2.67 -23.58

// Klumpp H, 2013

-30 -98 -4

28 -86 4

-64 -42 32

8 -16 52

// Kraus J, 2018

30 0 -16

// Thomaes K, 2012

-24 9 54

24 3 51

36 -18 -27

27 -45 -21

-36 3 9

3 12 48

-51 -36 33

51 6 39

-57 3 39

// Schwarzmeier H, 2019

33	-4	-17
45	-4	-11
-36	-25	-20
33	-4	-14
-30	-13	19
-3	-55	-26
-12	-64	-14
42	5	-5
-42	14	19
-30	41	-2

// Mazza M, 2012

49.2	-5.86	-3.03
-18.25	-14.15	-21.63

// Korgaonkar, 2021

-50	-78	-36
-40	-84	-44
68	-18	-28
48	-90	-16
-66	-58	-24
-66	-40	-28
-26	-38	-22

// Etkin, 2011

28	0	-28

// Etkin, 2011

-22	44	44
-22	48	12

30 62 14

// Yu, 2015

-60 -30 15

-60 -45 20

// Gaebler, 2013

3 21 9

12 24 12

3 15 -6

// Blair, 2012

-6 64 20

// Neumeister, 2018

63.13 -22.97 29.11

57.15 41.63 -2.96

50.67 15.36 -8.09

49.7 44.67 6.96

38.87 58.66 8

33.66 58.93 22.63

38.21 -9.33 27.06

49.65 -56.4 -17.78

45.26 -44.93 -21.08

40.54 -36.56 34.2

52.19 -36.05 16.02

-41.41 2.22 -28.75

-15.88 -97.62 4.22

31.52 -72.24 -2.46

19.92 -94.22 15.6

12.99 -18.81 -4.07

10.46 -21.52 -32.89

8.48	-29	-20.91
7.94	14.48	30.82
-6.96	-14.52	42.91
-12.58	8.99	30.6
10.13	30	35.98
23.48	31.45	-18.18
37.67	9.58	-11.78
-19.63	-5.18	-17.19
-34.38	-52.7	3.46
-42.17	-38.02	-11.31
-36.96	-9.91	-20.91

// Chechko, 2013

14	-86	12
34	-86	32
24	-4	-14
-60	-4	10
22	-2	-12
34	-86	32
-60	-4	10
14	-86	12
24	-4	-14
34	-86	32
14	-86	12
-60	-4	-10

// Mitterschiffthaler M T, 2008

-5	30	24
2	-60	44

// Ruhe H G, 2012

-26	4	12

-28	6	15
-12	-6	-6

// Frodl T, 2009

-42	6	38
-32	58	14

// Heitmann C Y, 2017

-38.38	14.91	11.85
-63.8	-46.58	17.3
5.96	-50.74	53.65
44.9	-21.55	-12.81
26.82	-28.46	-4.22
39.48	27.03	-3.44
48.01	23.66	-6.6
9.89	25.97	40.48
6.66	43.21	43.53
43.11	11.7	45.75

Appendix G: Mood Disorders Subgroup Foci (controls>patients)

// Reference=MNI

// Cerullo M A, 2016

-39.39 57.77 -32.44

18.36 108.01 -7.2

-38.7 -4.22 32.97

-55.13 -5.25 -20.28

-16.7 -2.11 -24.33

32.07 8.55 67.89

-13.65 25.95 -33.41

2.8 37.01 25.69

// Cerullo M A, 2016

-42.62 64.1 -32.93

-64.71 -1.63 -13.78

-26.17 -33.63 -18.23

-29.08 -60.14 10.79

-58.51 -.47 -40.72

// Cerullo M A, 2016

-39.39 57.77 -32.44

18.36 108.01 -7.2

-38.7 -4.22 32.97

-55.13 -5.25 -20.28

-16.7 -2.11 -24.33

-42.62 64.1 -32.93

-64.71 -1.63 -13.78

-26.17 -33.63 -18.23

32.07 8.55 67.89

-13.65 25.95 -33.41

2.8 37.01 25.69

-29.08 -60.14 10.79

-58.51 -.47 -40.72

// Arnone D, 2012

28 -88 25

-11 -81 5

32 -70 -10

14 -88 -5

-25 -84 30

32 -88 10

-42 7 45

39 -4 55

56 -32 -10

53 -70 0

35 -7 60

-11 -74 45

// Etkin, 2011

-10 28 -2

-4 40 -16

// Burger, 2017

−24 4 4

−52 −6 −4

2 46 26

−22 50 10

4 40 28

24 54 0

−20 42 24

4 48 26

4 40 28

26 54 -4

// Chechko, 2013

34	−54	−28
−48	18	22
50	28	14
46	−66	−12
10	−80	−22
−8	−84	−28
−2	6	70
40	−48	46
−28	−66	44
54	26	−4
48	-64	-14
-60	-4	-10
-60	-4	-10

// Ruhe H G, 2012

50	20	-6
-34	22	-6
42	16	27
48	-2	51
-54	16	0
-4	10	63
-42	-54	-21
-8	26	42
-6	-20	51
-4	10	63
50	20	-6
-34	22	-6
42	16	27
-42	-54	-21
46	-40	-24
-16	-38	-21

58	-6	-12
-12	-22	48
54	32	3
44	2	48
54	-48	12
48	-36	6
-40	-54	-18

Appendix H: Mood Disorders Subgroup Foci (patients>controls)

// Reference=MNI

// Cerullo M A, 2016

70.06 13.67 6.81

-23 51.69 11.38

-7.29 38.67 -34.55

-54.98 22.69 10.83

6.01 76.64 42.41

-32.09 -54.94 37.15

-45.56 40.5 -7.5

2.59 100.71 23.7

21.65 23.24 -30.24

41.3 1.32 14.86

-26.07 -6.2 6.23

// Cerullo M A, 2016

63.58 9.69 -2.82

-38.95 -13.39 -3.06

-.44 26.71 16.56

24.61 103.08 -30.28

18.43 19.79 -33.26

-45.56 89.88 11.78

3.13 -2.43 52.42

-26.57 51.25 -35.38

41.18 -13.17 -7.34

35.14 43.46 64.93

// Cerullo M A, 2016

70.06 13.67 6.81

-23 51.69 11.38

-7.29 38.67 -34.55

-54.98 22.69 10.83

6.01	76.64	42.41
-32.09	-54.94	37.15
-45.56	40.5	-7.5
2.59	100.71	23.7
21.65	23.24	-30.24
41.3	1.32	14.86
-26.07	-6.2	6.23
63.58	9.69	-2.82
-38.95	-13.39	-3.06
-.44	26.71	16.56
24.61	103.08	-30.28
18.43	19.79	-33.26
-45.56	89.88	11.78
3.13	-2.43	52.42
-26.57	51.25	-35.38
41.18	-13.17	-7.34
35.14	43.46	64.93

// Arnone D, 2012

56	-7	30
-14	-53	50
18	-56	-30

// Etkin, 2011

28	0	-28

// Etkin, 2011

-22	44	44
-22	48	12
30	62	14

// Chechko, 2013

14	-86	12
34	-86	32
24	-4	-14
-60	-4	10
22	-2	-12
34	-86	32
-60	-4	10
14	-86	12
24	-4	-14
34	-86	32
14	-86	12
-60	-4	-10

// Mitterschiffthaler M T, 2008

-5	30	24
2	-60	44

// Ruhe H G, 2012

-26	4	12
-28	6	15
-12	-6	-6

// Frodl T, 2009

-42	6	38
-32	58	14

Appendix I: Anxiety Disorders Subgroup Foci (controls>patients)

// Reference=MNI

// Blair K S, 2011

12.17	-98.96	7.44

// Kraus J, 2018

32	-2	-14

// Schwarzmeier H, 2019

24	29	37

42	-25	22

-42	-19	43

-60	-19	22

-54	-4	13

-57	-37	46

39	-4	40

-54	-34	-14

-3	29	61

-6	-52	34

-15	2	34

0	20	64

6	-73	43

45	-43	25

54	-43	31

39	11	37

// Mazza M, 2012

-11.46	-99.25	-7.88

// Wang, 2021

23.77	-45.85	73.5

13.65	36.45	58.81

11.39	27	49.7

// Korgaonkar, 2021

-18	-26	26
70	-52	36
-28	-58	-42
-12	-72	-46
10	-70	-48
-10	30	12
-30	-4	16
28	-68	-8
24	-56	-44
-20	-48	-26
-40	12	12
-24	30	28
-30	-52	-10
-18	4	36
-16	-60	48
-16	26	34
-24	40	-10

// Blair, 2012

-5	26	24
57	-33	21
-63	-42	31
-31	-58	44

// Blair, 2012

20	-7	-18

// Etkin, 2011

-10	28	-2

-4	40	-16

// Etkin, 2011

18	2	-16

// Yu, 2015

50	20	40

// Palm M E, 2011

33.14	29.9	-23.8

-47.83	34.39	-17.26

-8.96	46.43	-16.87

16.91	65.77	-18.11

-15.43	69.47	-11.2

-31.37	52.79	6.41

17.32	63.36	13.5

39.73	31.82	-15.13

-50.72	31.55	9.96

4.29	49.72	6.11

Anxiety Disorders Subgroup Foci (patients>controls)

// Reference=MNI

// Feldker K, 2017

2.28	-40.24	-2.95

19.48	-43.51	8.28

-9.55	-31.22	-9.15

27.02	-54.92	11.38

-49.2	9.07	-7.61

-39.58	-.73	-11.36

-64.02	7.18	10.56

-36.4	-8.63	-17.42

-50.09	5.49	15.01

// Kaldewaij R, 2019

6	54	6
6	62	14
38	-86	14
-40	-86	-20
-6	-56	18

// Blair K S, 2011

8.33	47.22	-14.82
-28.98	-2.67	-23.58

// Klumpp H, 2013

-30	-98	-4
28	-86	4
-64	-42	32
8	-16	52

// Kraus J, 2018

30	0	-16

// Thomaes K, 2012

-24	9	54
24	3	51
36	-18	-27
27	-45	-21
-36	3	9
3	12	48
-51	-36	33
51	6	39
-57	3	39

// Schwarzmeier H, 2019

33	-4	-17
45	-4	-11
-36	-25	-20
33	-4	-14
-30	-13	19
-3	-55	-26
-12	-64	-14
42	5	-5
-42	14	19
-30	41	-2

// Mazza M, 2012

49.2	-5.86	-3.03
-18.25	-14.15	-21.63

// Korgaonkar, 2021

-50	-78	-36
-40	-84	-44
68	-18	-28
48	-90	-16
-66	-58	-24
-66	-40	-28
-26	-38	-22

// Etkin, 2011

28	0	-28

// Etkin, 2011

-22	44	44
-22	48	12

30 62 14

// Yu, 2015

-60 -30 15

-60 -45 20

// Gaebler, 2013

3 21 9

12 24 12

3 15 -6

// Blair, 2012

-6 64 20

// Neumeister, 2018

63.13 -22.97 29.11

57.15 41.63 -2.96

50.67 15.36 -8.09

49.7 44.67 6.96

38.87 58.66 8

33.66 58.93 22.63

38.21 -9.33 27.06

49.65 -56.4 -17.78

45.26 -44.93 -21.08

40.54 -36.56 34.2

52.19 -36.05 16.02

-41.41 2.22 -28.75

-15.88 -97.62 4.22

31.52 -72.24 -2.46

19.92 -94.22 15.6

12.99 -18.81 -4.07

10.46 -21.52 -32.89

8.48	-29	-20.91
7.94	14.48	30.82
-6.96	-14.52	42.91
-12.58	8.99	30.6
10.13	30	35.98
23.48	31.45	-18.18
37.67	9.58	-11.78
-19.63	-5.18	-17.19
-34.38	-52.7	3.46
-42.17	-38.02	-11.31
-36.96	-9.91	-20.91

// Heitmann C Y, 2017

-38.38	14.91	11.85
-63.8	-46.58	17.3
5.96	-50.74	53.65
44.9	-21.55	-12.81
26.82	-28.46	-4.22
39.48	27.03	-3.44
48.01	23.66	-6.6
9.89	25.97	40.48
6.66	43.21	43.53
43.11	11.7	45.75
